# Supplementary material for: Metabolome and transcriptomics analyses reveal quality differences between Camellia tachangensis F. C. Zhang and C. sinensis (L.) O. Kunzte
Source: PLoS One. 2024 Dec 5;19(12):e0314595. doi: 10.1371/journal.pone.0314595 (PMC11620563; doi:10.1371/journal.pone.0314595)
Supplement: S1 Table — (DOC) [file pone.0314595.s001.doc]

Supplementary Table 1. Sample sequencing data evaluation statistical.

| **ID** | **Read Number** | **Base Number** | **GC Content** | **%≥Q30** |
| --- | --- | --- | --- | --- |
| DaChang1 | 21,656,178 | 6,459,460,570 | 45.24% | 94.83% |
| DaChang2 | 22,282,939 | 6,663,213,198 | 44.95% | 94.48% |
| DaChang3 | 20,415,766 | 6,104,229,792 | 44.94% | 93.65% |
| FDDB1 | 20,481,532 | 6,123,141,050 | 45.54% | 94.68% |
| FDDB2 | 23,999,972 | 7,178,030,836 | 45.54% | 94.27% |
| FDDB3 | 21,292,809 | 6,366,610,884 | 45.15% | 94.61% |

Note: %≥Q30 indicates the percentage of bases with Clean Data quality value greater than or equal to 30.
