# Supplementary material for: Metabolome and transcriptomics analyses reveal quality differences between Camellia tachangensis F. C. Zhang and C. sinensis (L.) O. Kunzte
Source: PLoS One. 2024 Dec 5;19(12):e0314595. doi: 10.1371/journal.pone.0314595 (PMC11620563; doi:10.1371/journal.pone.0314595)
Supplement: S2 Table — (DOC) [file pone.0314595.s002.doc]

Supplementary Table 2. Assembly result statistics

| **Length Range** | **Transcript** | **Unigene** |
| --- | --- | --- |
| 200-300 | 188,006(26.71%) | 0(0%) |
| 300-500 | 125,052(17.76%) | 13,281(25.23%) |
| 500-1000 | 132,704(18.85%) | 11,232(21.34%) |
| 1000-2000 | 147,353(20.93%) | 17,060(32.41%) |
| 2000+ | 110,851(15.75%) | 11,067(21.02%) |
| Total Number | 703,966 | 52,640 |
| Total Length | 737,893,576 | 71,943,102 |
| N50 Length | 1,838 | 1,907 |
| Mean Length | 1048.19 | 1366.7 |

Note: Length Range indicates the different length ranges of Unigene;The numbers in the table represent the number of Unigenes in the corresponding interval, and the percentages in parentheses represent the proportion of Unigenes in the corresponding length interval.
