# Supplementary material for: Metabolome and transcriptomics analyses reveal quality differences between Camellia tachangensis F. C. Zhang and C. sinensis (L.) O. Kunzte
Source: PLoS One. 2024 Dec 5;19(12):e0314595. doi: 10.1371/journal.pone.0314595 (PMC11620563; doi:10.1371/journal.pone.0314595)
Supplement: S3 Table — (DOC) [file pone.0314595.s003.doc]

Supplementary Table 3. Unigene annotation statistics

| **Anno_Database** | **Annotated_Number** | **300<=length<1000** | **length>=1000** |
| --- | --- | --- | --- |
| COG_Annotation | 9,052 | 1,892 | 7,160 |
| GO_Annotation | 26,954 | 7,949 | 18,997 |
| KEGG_Annotation | 20,860 | 5,289 | 15,571 |
| KOG_Annotation | 17,862 | 4,800 | 13,062 |
| Pfam_Annotation | 23,300 | 5,627 | 17,673 |
| Swissprot_Annotation | 19,827 | 4,630 | 15,197 |
| TrEMBL_Annotation | 32,335 | 9,631 | 22,704 |
| eggNOG_Annotation | 24,969 | 6,341 | 18,628 |
| nr_Annotation | 34,586 | 11,345 | 23,241 |
| All_Annotated | 35,323 | 11,858 | 23,457 |

Note: Annotated databases represent functional databases;Annotated_Number indicates the number of Unigenes annotated to the database;300≦ length < 1000 indicates the number of Unigenes whose length is greater than or equal to 300 and less than 1000 bases;1000 indicates the number of Unigenes greater than 1000 bases in length annotated to the database.
