# Supplementary material for: Metabolome and transcriptomics analyses reveal quality differences between Camellia tachangensis F. C. Zhang and C. sinensis (L.) O. Kunzte
Source: PLoS One. 2024 Dec 5;19(12):e0314595. doi: 10.1371/journal.pone.0314595 (PMC11620563; doi:10.1371/journal.pone.0314595)
Supplement: S4 Table — (DOC) [file pone.0314595.s004.doc]

Supplementary Table 4. Statistics of SSR analysis results

| **type** | **number** |
| --- | --- |
| c | 1,281 |
| c* | 23 |
| p1 | 7,216 |
| p2 | 7,316 |
| p3 | 2,758 |
| p4 | 222 |
| p5 | 32 |
| p6 | 49 |
| Total | 18,897 |

Note: c* indicates composite type SSR with overlapping position.
