# Supplementary material for: Metabolome and transcriptomics analyses reveal quality differences between Camellia tachangensis F. C. Zhang and C. sinensis (L.) O. Kunzte
Source: PLoS One. 2024 Dec 5;19(12):e0314595. doi: 10.1371/journal.pone.0314595 (PMC11620563; doi:10.1371/journal.pone.0314595)
Supplement: S5 Table — (DOC) [file pone.0314595.s005.doc]

Supplementary Table 5. The relationship between enzymes and related genes in the caffeine and theanine metabolic pathways of *C. sinensis* and *C. tachangensis.*

| **Gene name** | **Annotation** | **Number** | **ID** | **FPKM of *C. tachangensis*** | **FPKM of. *C.* sinensis** |
| --- | --- | --- | --- | --- | --- |
| GDH | Glutamate dehydrogenase | 2 | Unigene_082187 | 4.45 | 23.27 |
|  |  |  | Unigene_192408 | 18.47 | 9.02 |
| GOGAT | Glutamate synthase | 2 | Unigene_091374 | 5.31 | 43.65 |
|  |  |  | Unigene_087527 | 2.2 | 6.17 |
| PYCR | Pyrroline-5-carboxylate reductase | 1 | Unigene_192618 | 8.33 | 25.54 |
| AST | Aspartate aminotransferas | 2 | Unigene_101416 | 3.97 | 1.16 |
|  |  |  | Unigene_078464 | 55.56 | 135.85 |
| AK | Aspartokinase | 1 | Unigene_100269 | 0.99 | 6.29 |
| DAPDC | Diaminopimelate decarboxylase | 1 | Unigene_109048 | 40.06 | 17.06 |
| P5CR | Delta-1-pyrroline-5-carboxylate synthase | 1 | Unigene_199880 | 0 | 0.92 |
| Gys | Cysteine synthase | 3 | Unigene_097985 | 5.3 | 2.32 |
|  |  |  | Unigene_099235 | 33.42 | 1.37 |
|  |  |  | Unigene_091063 | 12.82 | 45.99 |
| SATase | Serine acetyltransferase | 2 | Unigene_100433 | 12.99 | 1.61 |
|  |  |  | Unigene_085000 | 129 | 33.18 |
| MetE | 5-methyltetrahydropteroyltriglutamate--homocysteine methyltransferase | 1 | Unigene_201955 | 0 | 2.76 |
| SHMT | Serine hydroxymethyltransferase | 2 | Unigene_154973 | 9.68 | 3.02 |
|  |  |  | Unigene_088537 | 434.90 | 1113.75 |

Note: 0 indicates that no expression is detected.
