# Supplementary material for: Metabolome and transcriptomics analyses reveal quality differences between Camellia tachangensis F. C. Zhang and C. sinensis (L.) O. Kunzte
Source: PLoS One. 2024 Dec 5;19(12):e0314595. doi: 10.1371/journal.pone.0314595 (PMC11620563; doi:10.1371/journal.pone.0314595)
Supplement: S6 Table — (DOC) [file pone.0314595.s006.doc]

Supplementary Table 6. The relationship between enzymes and related genes in the flavonoid metabolic pathways of *C. sinensis* and *C. tachangensis.*

| **Gene name** | **Annotation** | **Number** | **ID** | **FPKM of *C. tachangensis*** | **FPKM of. *C.* sinensis** |
| --- | --- | --- | --- | --- | --- |
| CHS | Chalcone synthase | 1 | Unigene_131139 | 664.55 | 97.16 |
| CHI | Chalcone isomerase | 1 | Unigene_186355 | 251.66 | 44.70 |
| ANS | Anthocyanidin Synthase | 1 | Unigene_125371 | 215.77 | 97.24 |
| DFR | Dihydroflavonol 4-reductase | 3 | Unigene_189931 | 174.1 | 23.42 |
|  |  |  | Unigene_101536 | 2.05 | 0 |
|  |  |  | Unigene_101707 | 184.93 | 70.84 |
| F3'5'H | Flavonoid-3',5'-hydroxylase | 1 | Unigene_196413 | 0.36 | 30.94 |
| CYP73A | Trans-cinnamate 4-monooxygenase | 1 | Unigene_148903 | 178.08 | 64.14 |

Note: 0 indicates that no expression is detected.
