# Supplementary material for: Metabolome and transcriptomics analyses reveal quality differences between Camellia tachangensis F. C. Zhang and C. sinensis (L.) O. Kunzte
Source: PLoS One. 2024 Dec 5;19(12):e0314595. doi: 10.1371/journal.pone.0314595 (PMC11620563; doi:10.1371/journal.pone.0314595)
Supplement: S7 Table — (DOC) [file pone.0314595.s007.doc]

Supplementary Table 7. Metabolite differences of *C. tachangensis* and *C. sinensis*.

| **Compounds** | **C. sinensis_Mean** | **C. tachangensis_Mean** | **Fold_**  **change** | **log_2_FC** | **Pvalue** | **VIP** | **regulated** | **KEGG_**  **annotation** | **CAS** |
| --- | --- | --- | --- | --- | --- | --- | --- | --- | --- |
| L-Serine | 2034579.22 | 586582.42 | 0.29 | -1.82 | 0.00101 | 1.37 | down | C00065 | 56-45-1 |
| L-Threonine | 2508810.70 | 631775.17 | 0.25 | -2.00 | 0.00198 | 1.39 | down | C00188 | 72-19-5 |
| L-Homoserine | 2562248.27 | 687258.33 | 0.27 | -1.90 | 0.01211 | 1.35 | down | C00263 | 672-15-1 |
| L-Asparagine | 570528.58 | 63305.29 | 0.11 | -3.21 | 0.00093 | 1.39 | down | C00152 | 70-47-3 |
| L-Lysine | 663949.81 | 183337.89 | 0.28 | -1.88 | 0.04973 | 1.24 | down | C00047 | 56-87-1 |
| O-Acetylserine | 141503.85 | 191079.72 | 1.35 | 0.43 | 0.02514 | 1.27 | up | C00979 | 5147-00-2 |
| L-Glutamic acid | 13887588.92 | 6106882.14 | 0.44 | -1.23 | 0.01108 | 1.28 | down | C00025 | 56-86-0 |
| L-Theanine | 1831670.21 | 697136.43 | 0.38 | -1.46 | 0.04345 | 1.18 | down | -- | 3081-61-6 |
| O-Phospho-L-serine | 62865.68 | 24694.78 | 0.39 | -1.53 | 0.02999 | 1.24 | down | C01005 | 17885-08-4 |
| Caffeine | 9542589.13 | 5129657.58 | 0.54 | -0.90 | 0.00824 | 1.33 | down | C07481 | 58-08-2 |
| Fustin | 8.56 | 1379989.14 | 161272.49 | 17.29 | 0.00644 | 1.39 | up | C01378 | -- |
| Epicatechin | 4359541.83 | 13624341.60 | 3.13 | 1.65 | 0.00006 | 1.40 | up | C09727 | 490-46-0 |
| Gallocatechin | 1564369.35 | 14504.67 | 0.01 | -6.78 | 0.00273 | 1.40 | down | C12136 | 970-74-1 |
| Epigallocatechin | 4404906.99 | 113790.61 | 0.03 | -5.36 | 0.00079 | 1.40 | down | C12136 | 970-74-1 |
| L-Aspartic Acid | 5552339.54 | 2079125.15 | 0.37 | -1.46 | 0.00431 | 1.33 | down | C00049 | 56-84-8 |
