# Supplementary material for: Metabolome and transcriptomics analyses reveal quality differences between Camellia tachangensis F. C. Zhang and C. sinensis (L.) O. Kunzte
Source: PLoS One. 2024 Dec 5;19(12):e0314595. doi: 10.1371/journal.pone.0314595 (PMC11620563; doi:10.1371/journal.pone.0314595)
Supplement: Attachment — Submitted filename: Response to Reviewers.docx [file pone.0314595.s009.docx]

Dear Reviewer,
We thank you for the opportunity to revise the manuscript “Metabolome and Transcriptomics Analyses Reveal Quality Differences between Camellia tachangensis F. C. Zhang and C. sinensis (L.) O. Kunzte” for publication in Plos one. We also thank the reviewers and you for the helpful comments. Following those suggestions, we have made several revisions to the manuscript as outlined below. We look forward to your response and hope the revisions will enable you to accept this manuscript version. In this revised version, changes to our manuscript were all highlighted within the document using red-colored text.

We also appreciate your clear and detailed feedback and hope the explanation fully addresses your concerns. In the remainder of this letter, we discuss your comments individually along with our corresponding responses.
To facilitate this discussion, we first retype your comments in italic font and then present our responses to the comments.

**Responses Reviewer #1**

**Comment 1:**  *Line 48: Space problem*

**Response 1:** Thank you for pointing out our issue. We have added a space between the last word of the sentence in this section and the citation. You can see our modification in line 49.

**Comment 2:** *55: C. tachangensis F. C. Zhang. The authority name should not italics.*

**Response 2:** Thank you for pointing out our issue. We have made modifications to this section, and the authoritative names are no longer italicized. You can see the changes in line 56.

**Comment 3-6:** *74: Space problem.*

*78: Same as above.*

*82: Same as above.*

*85: Same as above. There must be space between the term “breeding” and citation “[16]”.*

**Response 3-6:** Thank you for pointing out our issue. Regarding the issue with citation spacing, we have reviewed the entire article and added a space between the last word of sentences and the citations. You can see the changes in lines 74, 79, 83, and 86. All such issues in the manuscript have been corrected.

**Comment 7:** *102: No need to mention this subheading “Materials” here.*

**Response 7:** Thank you for bringing up this issue. We have deleted the subheading "Materials." You can see this change in line 102.

***Comment 8:*** *55-56: Why is this sentence italics?*

**Response 8:** Thank you for pointing out this issue. The error occurred due to our oversight, and we have now corrected the formatting of this sentence. You can see the changes in lines 56-57.

***Comment 9:*** *177: Multiple reaction monitoring (MRM). Capitalize the first letter of the last words.*

**Response 9:** Thank you for pointing out our error. We have now corrected this word, and you can see the change in line 177.

**Comment 10:** *Table 1: Keep the table in the center or on the left side.*

**Response 10:** Thank you for pointing out our error. We have corrected the table formatting and centered all the tables. You can see this change in line 203.

**Comment 11:** *274: among which? Or* *among them?*

**Response 11:** Thank you for pointing out the issue. We have changed "among them" to "among which" in this section. You can see this in line 282.

**Comment 12:** *335: Why the authors provide citations in the results section?*

**Response 12:** Thank you for raising this issue. The section on the accumulation of organic acids in the results made assumptions and provided literature support. We have moved this section to the discussion part and removed the references from the results section. You can see this change in lines 392-394 of the document.

**Comment 13:** *357: Same as above.*

**Response 13:** Thank you for pointing out this issue. The section in the results on the biosynthesis of flavonoids made assumptions and provided literature support. We have removed the references from this part of the results. You can see this change in line 352.

**Comment 14:** *Keep the space between the two lines in the caption of the figure as per journal guidelines.*

**Response 14:** Thank you for pointing out the issue. We have changed the formatting of all the captions to double spacing. You can see this change in lines 216-219, 231, 252-253, 290-292, 312-314, 336-341, and 358-362.

**Comment 15:** *295: No need to add a full stop in the subheading. Follow journal guidelines.*

**Response 15:** Thank you for pointing out the issue. We have reviewed all the subheadings and removed the periods from them. You can see this change in lines 293-294.

**Comment 16:** *339: Some text is bold…*

**Response 16:** Thank you for pointing out the issue. Regarding the bold font in the captions, we have changed it to regular font. You can see this change in lines 312, 336, and 358.

**Comment 17:** *389: “Qunfeng Zhang and colleagues (2018)[41]? No need to mention the year. It should be Qunfen**g Zhang and colleagues [41].*

**Response 17:** Thank you for raising these issues. We have made changes to this section and removed the year. You can see this in line 385.

**Comment 18:** *In the discussion section part please keep the space between the last word of the sentence and the citation. Previously I mention this problem, but the authors ignore…*

**Response 18:** Thank you for pointing out the issue. I have reviewed the entire text and added a space between the last word of each sentence and the citation. You can see these changes throughout the document.

**Responses Reviewer #2**

**Comment 19:** *The* *authors address my concernings already. Anyyway, both Camellia tachangensis and C. sinensis belong to Sect. Thea (L.) Dyer, so they both are coverntaional tea, the former is wild and tea the later is cultivated tea. The usage of genus name Camellia should be more careful, the second on in the text should be C..*

**Response 19:** Thank you for your question. We have checked the usage of genus name to *Camellia tachangensis* ad *C. sinensis* in the article. Except for the first occurrence where we used the full names all other instances have been changed to *C.*.

Thank you again for your positive comments and valuable suggestions to improve the quality of our manuscript. We want to take this opportunity to thank you for all your time involved and this great opportunity for us to improve the manuscript. We hope you will find this revised version satisfactory.

Sincerely,

Lunxian Liu, Xinglin Wang, Qiuying Li, Han Dai, Qihang Zhou, Xingpan Meng, Zhongting Chen, Ximin Zhang, Zhengdong Zhang, Yingliang Liu, Tie Shen and Yin Yi
